# Endoscopic resection of advanced ampullary adenomas: a single-center 14-year retrospective cohort study

**DOI:** 10.1007/s00464-018-6392-9

**Published:** 2018-08-23

**Authors:** Sophia E. van der Wiel, Jan-Werner Poley, Arjun D. Koch, Marco J. Bruno

**Affiliations:** Department of Gastroenterology and Hepatology, Erasmus MC, University Medical Center Rotterdam, Postbus 2040, 3000 CA Rotterdam, The Netherlands

**Keywords:** Ampulla of Vater, Ampullary adenoma, Endoscopic resection, Endoscopic ampullectomy, ERCP

## Abstract

**Background:**

Endoscopic ampullectomy has been recognized as a safe and reliable means to resect selective tumors of the ampulla of Vater and is associated with lower morbidity and mortality rates compared to surgical resection. Success rates range from 42 to 92%, with recurrences reported in up to 33%. Studies on endoscopic resection of advanced lesions such as those with intraductal extension of adenoma (IEA) and lateral spreading adenomas (LSA) are limited. We aimed to evaluate the technical success, complications, and recurrence of endoscopic resection of ampullary adenomas, including advanced lesions.

**Methods:**

All patients referred to the Erasmus Medical Center for endoscopic resection of an ampullary lesion were retrospectively identified between 2002 and 2016. Endoscopic success was defined as complete excision of the adenoma, irrespective of the number of attempts, in the absence of recurrence.

**Results:**

We included 87 patients with a median age of 65 years. Of these, 56 patients (64%) had an adenoma confined to the ampulla (ACA), 20 patients (23%) had an LSA, and 11 patients (13%) were treated for an IEA. The median lesion sizes were 24.6 mm, 41.4 mm, and 16.3 mm, respectively (*P* < 0.001). Complications occurred in 22 patients (25.3%), of which hemorrhage was most prevalent (12.6%), followed by perforation (8.1%). Complications were equally divided (*P* = 0.874). The median follow-up duration was 21.1 months (12–45.9) for ACA, 14.7 months (4.2–34.5) for LSA, and 5.8 months (3.7–22.0) for IEA (*P* = 0.051). Endoscopic resection was curative in 87.5% of patients with an ACA, 85% in patients with an LSA, and in only one patient with an IEA (*P* < 0.001). Recurrence occurred in 10 patients (11.5%) (*P* = 0.733).

**Conclusion:**

Endoscopic ampullectomy is safe and highly successful in selected patients with an adenoma with or without lateral spreading. Outcomes of endoscopic treatment adenomas with an intraductal extension are less favorable and in these cases surgery should be considered.

Lesions of the ampulla of Vater are relatively rare. Adenomas are the most common benign tumors arising from the ampulla even though benign neoplasms account for < 10% of all periampullary neoplasms [1]. The detection of ampullary adenomas has increased over the last years most likely due to the more abundant use of esophagogastroduodenoscopy and ultrasonography [2]. As in colorectal adenomas, ampullary adenomas can undergo malignant transformation, and therefore it is essential to completely remove the lesion [3]. Historically ampullary adenomas have been resected surgically [4, 5]. Over the last decades, endoscopic ampullectomy (EA) has been recognized as a safe and reliable alternative treatment for selective tumors of the ampulla of Vater [5–7]. EA has lower morbidity and mortality rates than surgical procedures [3, 8]. Success rates after EA have been reported within a wide range from 46 to 92% and are largely based on retrospective, heterogeneous case series. Studies have shown that multiple procedures may be required to completely remove adenomatous tissue, in particular for larger lesions [9–14]. It is difficult to compare the outcomes of the various studies due to the lack of a consistent definition of ‘success’ and highly variable follow-up length. Additionally, the success rate also appears to be dependent on the extent of the tumor, i.e., whether it is confined to the ampulla, laterally spreading beyond the ampulla over the duodenal surface or growing intraductally. The overall complication rate of EA is around 15% and mainly consists of bleeding and pancreatitis. Recurrence of adenomas is reported in up to 33% of the cases, despite supposedly complete removal of the tumor at the index procedure [10, 12, 15]. Despite the increasing number of studies concerning endoscopic resection of ampullary tumors, studies reporting on the outcome of resection of ampullary adenomas with lateral spreading or intraductal extension are limited. There seems to be consensus that every patient with an ampullary tumor should be given a chance of endoscopic resection as long as the tumor appears benign and tumor size is not a contraindication [9, 16]. The aim of our study was to evaluate the technical success, complications and recurrence of endoscopic resection ampullary adenomas, in particular lateral spreading ampullary adenomas and those with intraductal extension.

## Materials and methods

We conducted a retrospective study in patients referred to the Erasmus MC, University Medical Center Rotterdam (Rotterdam, The Netherlands) for endoscopic resection of an ampullary adenoma over a 14-year period (between January 2002 and November 2016). All 107 cases were identified using an electronic endoscopic database reporting system (ENDOBASE, Olympus, Hamburg) searching for the terms ‘papillary resection’, ‘papillectomy’, ‘ampullectomy’, ‘adenoma’, and ‘spreading’. Additionally, a search was done in the nationwide network and registry of histo- and cytopathology in the Netherlands (PALGA) to search for patients diagnosed with an ampullary adenoma at our institution. We identified 87 patients with ampullary tumors that were histologically confirmed to be adenomas. Pathology slides were not re-examined. Additional inclusion criteria included adenomas of both major papilla and minor papilla, without invasive cancer on biopsy, and adenomas with substantial intraductal extension and adenomas with a lateral spreading growth pattern. A lateral spreading adenoma was defined as an adenoma of ≥ 10 mm in diameter that extends laterally along the surface of the gastrointestinal tract [17]. Patients with FAP were included in our study. Non-adenomatous tumors of the ampulla of Vater (neuroendocrine neoplasms, carcinomas) were excluded. Data that were extracted from the electronic patient records included patient demographics, clinical presentation, laboratory results, diagnostic findings, details on the endoscopic resection, follow-up, and morbidity and mortality.

The decision to perform endoscopic ultrasound (EUS) prior to endoscopic retrograde cholangiopancreatography (ERCP) was at the discretion of the treating physician and endoscopist. In early years not always performed, but EUS evaluation has become part of routine work-up during the last years. Endoscopic resection was performed using a side-viewing therapeutic duodenoscope. Procedures were done under either conscious sedation, anesthesia administered propofol sedation or general anesthesia. Rectal NSAID’s were administered during the procedure since 2010 to reduce the risk of pancreatitis. The technique of EA is not standardized and dependent on local anatomy, extension and characteristics of the adenoma and personal preference of the endoscopist. For snare resection, “ENDO CUT Q” mode was used with standard settings for polypectomy: effect 3, cut duration 1, cutting interval 6 (VIO200D, ERBE, Tübingen, Germany), and standard materials were used, among which an oval snare (Acusnare, Cook Medical) and a stiff hexagonal snare (Captivator, Boston Scientific, USA). In general, in cases without intraductal extension, the first step is cannulation of the pancreatic duct to fill the duct (partially) with diluted methylene blue to facilitate cannulation of the pancreatic duct after resection. In bulky or smaller adenomas in which en bloc resection is attempted, the caudal and lateral parts of the lesion are lifted with saline. This step is done carefully since “over-lifting” can lead to a more difficult resection of the ampulla itself. After resection, the specimen is retrieved with either the snare or a Roth-net and sent for pathological examination. At this stage, procedural bleeds are most likely to occur and these can be treated endoscopically with either adrenalin injection, a coag-grasper or clips. Visible residual adenomatous tissue is either resected with the snare or treated with argon plasma coagulation (APC). The final step of the procedure is cannulation of the pancreatic duct and placement of a 4 or 5 french unflanged single pigtail endoprosthesis. A plain abdominal film was obtained within 2 weeks after the resection to check for spontaneous stent migration. If no spontaneous migration had occurred, the stent was removed at gastroscopy. Lateral spreading lesions were removed in a piecemeal fashion with continuous lifting with gelofusine, methylene blue and diluted epinephrine (5 ml 1:10,000 in 500 ml gelofusine). In most instances, resection was started at the most caudal part of the lesion and the ampullary region itself was resected last. Resected pieces of the adenoma were positioned in either bulb or stomach and retrieved at the end of the procedure. In case of intraductal extension, a biliary and/or pancreatic sphincterotomy was performed to facilitate removal of intraductal tissue after a balloon sweep of the duct.

The surveillance protocol after treatment consists of a repeat examination after 1–6 month (depending on the initial success of treatment) followed by repeat examinations every 3–6 months for 2 years, and yearly thereafter for a total period of 5 years. Median follow-up time was calculated in months from the initial EA up to the most recent endoscopic examination or surgical intervention. Endoscopic success was defined as complete excision of the adenoma, disregarding the number of sessions needed, and the absence of recurrence over the total follow-up period. It was decided to use endoscopic success as an alternative for curative resection, because a R0 resection is often not acquired and in a number of cases complete removal is achieved in more than one session.

All statistical analyses were performed using SPSS 21.0 software (IBM Corp: Armonk, NY, USA). Data were expressed as mean ± standard deviation, median, and range. Statistical analysis included the Chi-square test, Fisher’s exact test, and Kruskal–Wallis test with *P* values < 0.05 regarded as significant. Survival analysis was demonstrated using the Kaplan–Meier method.

## Results

### Patient demographics and tumor characteristics

**Fig. 1:**
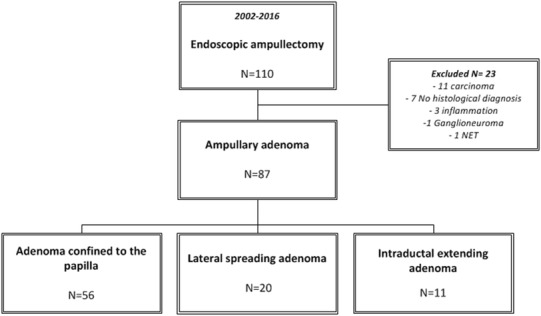
Study overview

**Fig. 2:**
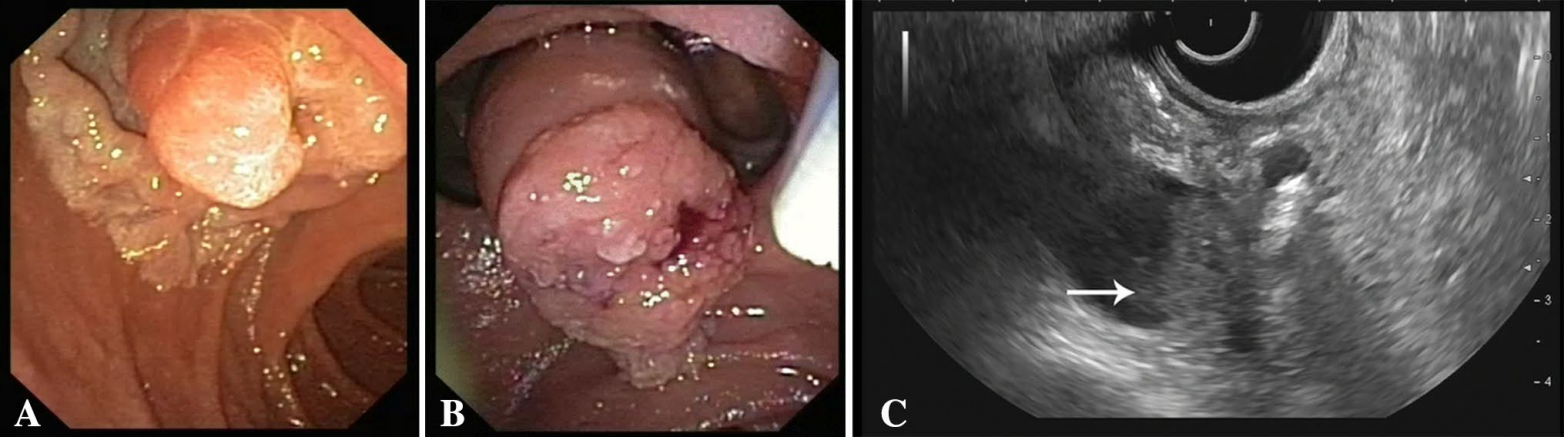
Overview of advanced ampullary adenomas. **A** LSA. **B** Intraductal extended adenoma with extension in the common bile duct. **C** Radial EUS image of the intraductal extended adenoma depicted in **B**

A total of 110 patients were treated endoscopically for suspected adenomas of the ampulla of Vater during the 14-year study period. Twenty-three cases were excluded from the study, because they did not meet the inclusion criteria (carcinoma [*n* = 11], non-availability of the resection specimen [*n* = 7], specimen showing signs of inflammation without dysplasia [*n* = 3], ganglioneuroma [*n* = 1], neuroendocrine tumor [*n* = 1]). Eventually, 87 patients were included, 60 patients (69%) with low-grade dysplasia (LGD) and 27 patients (31%) with high-grade dysplasia (HGD). Based on the anatomical extension of the adenoma, the 87 patients were divided into three groups: 56 patients had an adenoma confined to the ampulla (ACA), 20 patients had a lateral spreading ampullary adenoma (LSA), and 11 patients had an adenoma with intraductal extension (IEA). A study overview is depicted in Fig. 1. Figure 2 illustrates advanced ampullary adenomas. Patients demographics are listed in Table 1. A total of 45 men and 42 women with a mean age of 65 years (range 32–89 years) were included. In 18 patients (20.7%), the adenoma was found incidentally or during endoscopic examination in case of FAP. The remaining patients had symptoms for which medical investigations were initiated: abdominal pain in 34 patients (39.1%), anemia in 16 patients (18.4%), weight loss in six patients (6.9%), jaundice in four patients (4.6%), and pancreatitis in three patients (3.4%). No statistical differences were observed among groups. Tumor characteristics are listed in Table 2. Seventy-one (81.6%) patients underwent biopsy before EA: no dysplasia was seen in three patients (3.4%), 44 patients (50.6%) had LGD, and 24 patients (27.6%) had HGD. Post-ampullectomy histological diagnosis confirmed LGD in 60 patients (69%) and HGD in 27 patients (31%). The average tumor size was 27.7 mm (SD ± 15.9). LSA were significantly larger (41.4 mm, SD ± 12.9, *P* < 0.001).

**Table 1:** Demographics and clinical presentation

	Adenoma confined to the ampulla	Lateral spreading adenoma	Intraductal extending adenoma	Total	*P*
No. of patients, *n* (%)	56 (64.4%)	20 (23.0%)	11 (12.6%)	87	
Male, *n* (%)	29 (51.8%)	9 (43.9%)	7 (63.6%)	45 (51.7%)	0.610
Mean age (years)^a^	63.0 (13.3)	64.6 (11.7)	74.7 (10.7)	64.9 (13.1)	0.017
FAP, *n* (%)	7 (12.5%)	5 (25%)	0	12 (13.8%)	0.139
Presentation, *n* (%)					
Incidental	5 (8.9%)	2 (10%)	0	7 (8.0%)	0.859*
FAP follow-up	7 (12.5%)	4 (20%)	0	11 (12.6%)	0.334*
Biliary-pancreatic symptoms	9 (16.1%)	1 (5%)	2 (18.1%)	12 (13.8%)	0.377*
Abnormal laboratory results	5 (8.9%)	2 (10%)	2 (18.1%)	9 (10.3%)	0.497*
Non-specific symptoms	29 (51.8%)	11(55%)	6 (54.5%)	46 (52.9%)	0.948
Clinical symptoms, *n* (%)**					
Asymptomatic	16 (28.6%)	7 (35%)	2 (18.2%)	25 (28.7%)	0.698
Jaundice	2 (3.6%)	0	2 (18.2%)	4 (4.6%)	0.108*
Abdominal pain	22 (39.3%)	6 (30%)	6 (54.5%)	34 (39.1%)	0.290
GI bleeding	6 (10.7%)	1 (5.0%)	0	7 (8.0%)	0.595*
Anemia	8 (14.3%)	7 (35%)	1 (9.1%)	16 (18.4%)	0.126*
Pancreatitis	3 (5.4%)	0	0	3 (3.4%)	0.700
Cholangitis	2 (3.6%)	0	1 (9.1%)	3 (3.4%)	0.439*
Cholecystitis	2 (3.6%)	0	0	2 (2.3%)	1.000*
Weight loss	4 (7.1%)	1 (5.0%)	1 (9.1%)	6 (6.9%)	1.000*

*Fisher’s Exact test [Exact Sig. (2-sided)]

**Some patients had multiple complaints at clinical presentation

^a^Data are expressed as mean and standard deviation

**Table 2:** Tumor characteristics

	Adenoma confined to the ampulla *N* = 56	Lateral spreading adenoma *N* = 20	Intraductal extending adenoma *N* = 11	Total *N* = 87	*P*
Pre-resection biopsy, *n* (%)	45 (80.4%)	16 (80%)	10 (90.9%)	71 (81.6%)	0.841*
No dysplasia	3 (5.4%)	0	0	3 (3.4%)	0.737*
LGD	28 (50%)	11 (55%)	5 (45.5%)	44 (50.6%)	0.737*
HGD	14 (25%)	5 (25%)	5 (45.5%)	24 (27.6%)	0.737*
EUS assessment, *n* (%)	47 (83.9%)	13 (65%)	11 (100%)	71 (81.6%)	0.047*
Type of resection, *n* (%)					
En bloc	37 (66.1%)	1 (5.0%)	3 (27.3%)	41 (47.1%)	< 0.001
Piecemeal	18 (32.1%)	16 (80%)	8 (72.7%)	42 (48.3%)	< 0.001
Tumor size, in mm^a^	24.6 (15.1)	41.4 (12.9)	16.3 (4.3)	27.7 (15.9)	< 0.001
Histology resection specimen, *n* (%)					
LGD	39 (69.6%)	13 (65.0%)	8 (72.7%)	60 (69.0%)	0.835
HGD	17 (30.4%)	7 (35.0%)	3 (27.3%)	27 (31.0%)	0.835

*Fisher’s Exact test [Exact Sig. (2-sided)]

^a^Data are expressed as mean and standard deviation

### Endoscopic ampullectomy

Overall, success resection with absence of recurrence was achieved in 67 patients (77%); 87.5% for ACA, 85% for lateral spreading adenoma, and only 9.1% in case of intraductal extension (*P* < 0.001). Multiple procedures were required to successfully remove the adenoma in three patients with an ACA, seven patients with LSA and one patient with an IEA. En bloc resection was achieved in 37 patients with an ACA (66.1%). Post-ampullectomy APC application to treat remnant adenomatous tissue was applied in 58 patients (66.7%). EA was complemented with placement of a pancreatic duct stent in 60 patients (68.9%). Eight patients (9.2%) were referred for surgery after failed endoscopic resection, of whom six with intraductal extension. Of these latter patients, the final histopathological diagnosis was LGD (*n* = 3), HGD (*n* = 2) and invasive carcinoma (*n* = 1).

### Complications

Complications occurred in 22 patients (25.3%). The most common complication was post procedural hemorrhage (12.6%). Five patients required transfusions and seven patients underwent endoscopic management (adrenaline injection and/or hemoclip placement). In one patient, bleeding was controlled by coiling the gastroduodenal artery. (Retro)peritoneal perforation occurred in seven patients (8.1%). One patient developed a pneumothorax for which a thorax drain was placed. All other patients were treated with antibiotics only. Acute pancreatitis developed in three patients (3.4%), mild in two patients and severe in one patient. All three patients suffering from post-ERCP pancreatitis successfully underwent pancreatic duct stent placement. Cholangitis occurred in only one patient, treated with antibiotics. There was no procedure-related mortality. No stenosis of the papilla of Vater was observed in our cohort. There were no statistical significant differences in the occurrence of complications between groups. Endoscopic success rates and complications are shown in Table 3.

**Table 3:** Endoscopic success and post-procedural complications

	Adenoma confined to the ampulla *N* = 56	Lateral spreading adenoma *N* = 20	Intraductal extending adenoma *N* = 11	Total *N* = 87	*P*
Endoscopic success, *n* (%)	49 (87.5%)	17 (85.0%)	1 (9.1%)	67 (77.0%)	< 0.001*
Referral to surgery after failed ER, *n* (%)	1 (1.8%)	1 (5%)	6 (54.5%)	8 (9.2%)	< 0.001*
Complications, *n* (%)	15 (26.8%)	4 (20.0%)	4 (36.4%)	23 (26.4%)	0.630
Bleeding	8 (14.3%)	2 (10.0%)	1 (9.1%)	11 (12.6%)	0.823
Perforation	3(5.4%)	2 (10.0%)	2 (18.2%)	7 (8.1%)	0.337
Pancreatitis	3 (5.4%)	0	0	3 (3.4%)	0.423
Cholangitis	1 (1.8%)	0	0	1 (1.1%)	0.756
Papillary stenosis	0	0	0	0	–

*Fisher’s Exact test [Exact Sig. (2-sided)]

### Follow-up and recurrence

**Fig. 3:**
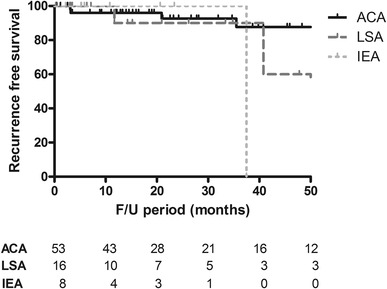
Recurrence-free survival according to endoscopic resection and extension of the adenoma

The median follow-up period was 18.6 months (IQR 7.6–39.5 months); 21.1 months in ACA, 14.7 months in LSA, and 5.8 months in IEA. Recurrence was observed in ten patients (10.7%). Five patients with ACA showed recurrence (8.9%), of the 20 patients treated for an LSA, four patients showed recurrence (20%). In the IEA only, 11 patients were endoscopically treated; six patients were referred for surgery, and in two patients it was decided to discontinue follow-up because of other medical conditions. Of the remaining three patients, one developed recurrence of the IEA. In only one patient with ACA (1.8%) surgical resection because of recurrence was required. LSA patients with recurrence were all treated endoscopically. The IEA patient with recurrence was also referred for surgery. After 2 years of follow-up, 93% of patients with an ACA were free from recurrence and 90% of patients with an LSA, as depicted in Fig. 3. Details are listed in Table 4.

**Table 4:** Follow-up and recurrence

	Adenoma confined to the ampulla *N* = 56	Lateral spreading adenoma *N* = 20	Intraductal extending adenoma *N* = 11	Total *N* = 87	*P*
Follow-up, months^a^	21.1 (12.0–45.9)	14.7 (4.2–34.5)	5.8 (3.7–22.0)	18.6 (7.6–39.5)	0.051
Recurrence, *n* (%)	5 (8.9%)	4 (20%)	1 (9.1%)	10 (11.5%)	0.305*
Time to recurrence, months^a^	9.2 (4.2–25.7)	21.8 (5.2–79.8)	21	13.1 (4.6–33.1)	0.733
Recurrence-free survival after 24 months	93%	90%	0%	–	–

*Fisher’s Exact test [Exact Sig. (2-sided)]

^a^Data are expressed as median and interquartile range

## Discussion

This is a retrospective single-center cohort study describing the endoscopic management and outcome of patients with (advanced) ampullary adenomas. Since the first large cohort study in 1993 by Binmoeller et al. [11], various reports have been published showing promising results of the endoscopic treatment of ampullary adenomas as an alternative for surgical resection [9, 12, 13, 18–21]. Our data show that EA is indeed a safe and effective treatment, also in patients with lateral spreading adenomas. In patients with intraductal extension however, surgical resection should be considered as primary treatment.

Currently, guidelines on the endoscopic or surgical management of ampullary adenomas are lacking. Literature data suggest that surgery is indicated for patients with larger lesions, for cases when no skilled interventional endoscopists with experience in ampullectomy are available and, obviously, in lesions suspected for malignancy and potential lymph node invasion [18, 22]. Surgical options include transduodenal ampullectomy and pancreaticoduodenectomy, but are associated with high morbidity and mortality rates. Morbidity rates vary from 4 to even 68% of patients who underwent pancreaticoduodenectomy and mortality is reported up to 7% [23, 24]. With the introduction of EA in 1983 by Suzuki et al. [25], treatment has shifted towards minimal invasive endoscopic resection as an alternative to surgery and this shift has been accelerated due to technical improvements in endoscopy over the last decades.

The study by Binmoeller et al. [11] included 25 patients with ampullary adenomas that were endoscopically treated, demonstrating lower morbidity and mortality rates than surgical intervention with a success rate of 75%. To date, outcome data of EA are largely based on retrospective case series in which success is reported in the range of 46–92% [9–14]. This wide range is explained by differences in selection criteria, tumor size, extent of the tumor, and probably of key importance, the experience of the endoscopist. In our study, the overall success rate of endoscopic resection was 77%. Categorization of adenomas based on the extent of the tumor, however, showed a significant difference in success rates. The success rate in patients with an adenoma confined to the ampulla and patients with a lateral spreading adenoma is excellent, 87.5% and 85%, respectively. In contrast, however, the endoscopic management of patients with an IEA was much less favorable with complete removal of the adenoma in 1 out of 11 patients only. Extension into the biliary duct or pancreatic duct of an ampullary adenoma has been historically regarded as a contraindication of endoscopic management [12, 13, 19, 26]. Therefore, studies evaluating the endoscopic management of IEAs are rare and mostly based on small groups. Bohnacker et al. [20] reported a success rate of 46% in 31 patients endoscopically treated for an IEA. The authors could not identify criteria predicting a successful resection and postulate that limited intraductal involvement allows for a reasonable attempt of endoscopic management, provided by experienced endoscopists. Cheng et al. [10] treated two patients with IEA endoscopically of which one was lost to follow-up and one showed no recurrence at 1-year follow-up. The optimal treatment strategy in patients with ampullary adenomas with intraductal extension remains elusive. Ideally, after appropriate ampullary resection and sphincterotomy, the intraductal extension of the adenomatous lesion is exposed, visually inspected, and removed. The role of radiofrequency ablation to treat intraductal extension of ampullary adenomas is currently under investigation and shows some promise [27, 28]. Needless to state that adequate follow-up is pivotal importance in order to timely revert to surgical resection not losing out on an opportunity for curative treatment.

Even though various studies have confirmed a decrease in procedure-related complications for endoscopic management of ampullary adenomas in comparison to surgical resection, complication rates are described up to 33% and remain as an important concern [10, 12, 15]. Catalano et al. [12] performed a large study combining the results of EA from four pancreaticobiliary endoscopy centers, including 103 patients with a success rate of 80% and a complication rate of 10%. In 2013, Onkendi et al. [8] published the results of a large comparative study of the outcomes of operative and endoscopic resection showing post-endoscopic complications in 29% of treated patients. Procedure-related complications in our cohort, including mainly bleeding, perforations and pancreatitis, occurred in 25.3% of patients with no statistical between groups.

Post EA bleeding was seen most often in our cohort (12.6%), with literature data indicating a median risk of 8.5% of cases [29]. Most patients were treated endoscopically, but one patient required coiling of the gastroduodenal artery to control the bleed. Although the IEAs and adenomas with a lateral growth pattern are reported to be associated with a higher bleeding risk, we did not observe this in our series [15, 20, 30].

Post-ERCP pancreatitis is the most common complication after EA, with an incidence reported between 8 and 19% [10, 12, 20, 30, 31]. Pancreatic stent placement during the procedure may reduce the risk of this complication [9, 20, 32, 33], as well as administration of nonsteroidal inflammatory drugs (NSAIDs) [34]. Previous studies reported a success rate of pancreatic stent placement in patients with an ampullary adenoma of 4–92% [11, 12, 35]. In the present study, pancreatic stent placement was successful in 69% of patients. Post-ERCP pancreatitis was diagnosed in only 3.4% of patients. Administration of rectal NSAIDs has become standard practice at our unit since 2010, in accordance with the ESGE guideline [36]. All three patients that suffered from post-ERCP pancreatitis in our series had undergone prophylactic pancreatic duct stent placement.

Perforation occurred in seven patients (8.1%). There were no statistical differences among groups (*P* = 0.337), however, as expected, our data indicate that perforation occurred more in the advanced adenoma groups. The incidence of perforation is higher in our cohort compared to previous series; however, patients were successfully managed with conservative treatment.

Papillary stenosis is a known late complication of EA with an incidence of 2.9–8%. No cases of papillary stenosis were reported in our cohort.

The median follow-up of patients with an ampullary adenoma treated endoscopically reported in literature ranges from 9 to 66 months with recurrence described up to 33% of cases [10, 12, 19, 20]. In our study, the median follow-up duration was 18.6 months. No clear guidance regarding the appropriate length of endoscopic follow-up is available, but several studies indicate a period of at least 2 years [9–12]. In the present study, recurrence occurred in 11.5% of patients after a median of 13.1 months (IQR 4.6–33.1), but in one case recurrence was found 55 months after initial therapy.

There are several potential limitations to our study. The retrospective nature makes this study prone to selection and recall bias. However, due to the rarity of this condition a prospective study is unlikely to be carried out. Also, the number of patients in the advanced adenoma groups was small making statistical comparisons between groups of limited value.

In conclusion, this single-center retrospective cohort study confirms that EA can be a safe and successful treatment modality for patients with an ampullary adenoma confined to the ampulla, but also for patients with a lateral spreading papillary adenoma. Meticulous endoscopic follow-up to detect and treat recurrence is pivotal. In case of intraductal extension of adenomatous tissue, endoscopic success rates are reduced to such a level that surgical resection should be considered.

## Abbreviations

APC: Argon plasma coagulation
ACA: Adenoma confined to the ampulla
EA: Endoscopic ampullectomy
ERCP: Endoscopic retrograde cholangiopancreatography
EUS: Endoscopic ultrasound
IEA: Intraductal extending adenoma
FAP: Familial adenomatous polyposis
HGD: High-grade dysplasia
LGD: Low-grade dysplasia
LSA: Lateral spreading adenoma
NSAIDs: Nonsteroidal inflammatory drugs
